# Mortality Statistics, Chicago

**Published:** 1875-03

**Authors:** 


***Chicago Mortality Report for January, 1875.*** Reported by Dr. BEN. C. MILLER, Sanitary Superintendent.

| | | | |
|---|---|---|---|
| Accident, by burns | 1 | Asphyxia | 1 |
| " crushed | 1 | Asthma | 4 |
| " by explosion | 2 | Ascitis | 2 |
| " by fall | 2 | Atelectasis pulmonum | 3 |
| " by rupture | 1 | Bowels, intussusception of | 1 |
| " by poisoning | 1 | Brain, abscess of | 1 |
| " by fracture of spine | 1 | " congestion of | 12 |
| " by suffocation | 1 | " inflammation of | 1 |
| " by scalding | 1 | " softening of | 2 |
| " by railroad | 5 | Bronchitis | 4 |
| Aphthæ | 1 | " capillary | 12 |
| Apoplexy | 7 | Caries of vertebræ | 1 |
| Arachnitis | 1 | Caries exanthema | 1 |

| | |
|---|---|
| Catarrh pulmonum | 1 |
| Cancer of breast | 2 |
| " of knee | 1 |
| " of stomach | 6 |
| " of uterus | 1 |
| Child birth | 2 |
| Cholera infantum | 1 |
| Consumption | 59 |
| Convulsions | 85 |
| Croup | 8 |
| " membranous | 3 |
| Cyanosis | 2 |
| Debility, general | 11 |
| Delirium tremens | 1 |
| Diabetes | 1 |
| Diarrhœa | 5 |
| " chronic | 1 |
| Diphtheria | 10 |
| Dropsy, general | 11 |
| Dysentery | 1 |
| Embolism | 2 |
| Enteritis | 9 |
| Epilepsy | 1 |
| Exposure | 4 |
| Erysipelas | 5 |
| Exhaustion | 1 |
| Fever, puerperal | 8 |
| " scarlet | 7 |
| " typhoid | 11 |
| Gangrene | 1 |
| Gastritis | 3 |
| Hemorrhage, umbilical | 2 |
| Heart, organic disease of | 2 |
| " hypertrophy of | 2 |
| " valvular disease of | 2 |
| " paralysis of | 1 |
| Hepatitis | 1 |
| Hip disease | 1 |
| Hernia, strangulated | 1 |
| Hydrocephalus | 6 |
| Inanition | 6 |
| Intemperance | 6 |
| Icterus | 1 |
| Ileus | 1 |
| Influenza | 1 |
| Jaundice | 1 |
| Kidneys, disease of | 1 |
| " Bright's disease of | 1 |
| " inflammation of | 2 |
| Laryngitis | 2 |
| Leucocythemia | 1 |
| Liver, cirrhosis of | 2 |
| " inflammation of | 1 |
| " apoplexy of | 1 |
| Lungs, congestion of | 10 |
| " hemorrhage of | 2 |
| " paralysis of | 1 |
| Metro peritonitis | 1 |
| Measles | 5 |
| Meningitis | 4 |
| " cerebro-spinal | 3 |
| " tubercular | 2 |
| Old age | 16 |
| Paralysis | 6 |
| Pericarditis | 1 |
| Peritonitis | 1 |
| " puerperal | 2 |
| Pelvic cellulitis | 1 |
| Pneumonia | 53 |
| " typhoid | 9 |
| Pneumo thorax | 1 |
| Plebitis uterine | 1 |
| Pleurisy | 2 |
| Pyæmia | 2 |
| Rheumatism | 2 |
| " inflammatory | 1 |
| Sarcoma | 1 |
| Scrofula | 3 |
| Septicæmia | 1 |
| " puerperal | 1 |
| Spine and brain inflammation | 1 |
| Stomach hemorrhage | 2 |
| " congestion | 1 |
| Syphilis | 4 |
| Suicide, arsenic | 1 |
| " hanging | 3 |
| " cutting throat | 1 |
| " shooting | 1 |
| Syncope following child birth | 1 |
| Tabes mesenterica | 13 |
| Manslaughter | 2 |
| Teething | 3 |
| Tetanus | 1 |
| Trismus | 2 |
| Urine, retention of | 1 |
| Uræmia | 1 |
| Uterus, hemorrhage of | 2 |
| Worms | 1 |
| Whooping cough | 1 |
| Womb and bowels, congestion of | 1 |
| Total | 568 |

Premature births, 7; Still births, 54. Total, 61.

COMPARISON.

Deaths in January, 1875, 568; in December, 1874, 455. Increase, 113. Deaths in January, 1874, 535. Increase, 33.

AGES.

| | | | |
|---|---|---|---|
| Under one year | 186 | Thirty years to forty | 61 |
| One year to two | 29 | Forty " " fifty | 58 |
| Two years to three | 31 | Fifty " " sixty | 39 |
| Three " " four | 10 | Sixty " " seventy | 32 |
| Four " " five | 10 | Seventy " " eighty | 20 |
| Five " " ten | 16 | Eighty " " ninety | 11 |
| Ten " " twenty | 20 | Ninety " " one hundred | 3 |
| Twenty " " thirty | 42 | Total | 568 |
| Males | 304 | Colored | 12 |
| Females | 264 | White | 556 |
| Total | 568 | Total | 568 |

Married, 214; Single, 354. Total, 568.

NATIVITIES.

| | | | | | |
|---|---|---|---|---|---|
| Austria | 1 | Denmark | 4 | Norway 10 N'foun'd 1 | 11 |
| Bohemia | 2 | England | 12 | Scotland | 5 |
| Canada | 2 | France | 1 | Sweden | 5 |
| Native—Chicago | 76 | Germany | 76 | Switzerland | 1 |
| Foreign, " | 187 | Ireland | 79 | Unknown | 14 |
| U. States, other parts | 91 | Italy | 1 | Total | 568 |

Deaths daily, 17½. Mean thermometer, 19°. Rain fall, .96 inches.

MORTALITY BY WARDS.

| Wards. | No. Deaths. | Pop. in 1874. | Percentage. |
|---|---|---|---|
| 1 | 4 | 5,725 | one death in 1,431 |
| 2 | 7 | 4,830 | " " 690 |
| 3 | 22 | 14,861 | " " 675 |
| 4 | 14 | 15,361 | " " 1,097 |
| 5 | 20 | 20,078 | " " 1,004 |
| 6 | 51 | 35,916 | " " 704 |
| 7 | 50 | 31,722 | " " 634 |
| 8 | 39 | 29,143 | " " 748 |
| 9 | 35 | 31,654 | " " 904 |
| 10 | 25 | 17,385 | " " 695 |
| 11 | 23 | 14,022 | " " 610 |
| 12 | 17 | 16,792 | " " 988 |
| 13 | 23 | 17,892 | " " 778 |
| 14 | 20 | 16,720 | " " 836 |
| 15 | 80 | 45,545 | " " 569 |
| 16 | 24 | 21,922 | " " 913 |
| 17 | 14 | 20,777 | " " 1,484 |
| 18 | 22 | 21,392 | " " 972 |
| 19 | 5 | 4,677 | " " 935 |
| 20 | 9 | 8,995 | " " 999 |
| | 504 | Ratio of deaths to population in 1874, one death in 870½. | |

| | | | |
|---|---|---|---|
| No. deaths in Wards | 504 | Manslaughter | 2 |
| Accidents | 17 | Mercy Hospital | 5 |
| Barge | 1 | Police Station | 2 |
| Hospital County | 9 | St. Luke's Hospital | 2 |
| Foundlings' Home | 16 | Suicides | 6 |
| Hospital Alexian Bros. | 3 | | |
| Home for Friendless | 1 | Total | 568 |

